# Addressing the Importance of Mental Health for Children from Refugee Backgrounds in the United States

**DOI:** 10.3390/children13101390

**Published:** 2026-10-13

**Authors:** Roxana Khalili, Maryan Toma, Keymia Ghodrati, Isain Zapata, Jean Bouquet

**Affiliations:** 1 College of Osteopathic Medicine, Rocky Vista University, Englewood, CO 80112, USA; 2 Department of Biomedical Sciences, Rocky Vista University, Englewood, CO 80112, USA; 3 Office of Research and Scholarly Activity, Rocky Vista University, Englewood, CO 80112, USA; 4 Department of Primary Care, Rocky Vista University, Englewood, CO 80112, USA

**Keywords:** children from refugee backgrounds, mental health, post-traumatic stress disorder, mental health intervention, trauma-informed practices

## Abstract

**Highlights:**

**What are the main findings?**

- Children from refugee backgrounds in the U.S. face elevated mental health challenges and significant barriers to care.
- School-based, trauma-informed peer mentorship programs may improve resilience, belonging, and well-being.

**What are the implications of the main findings?**

- Schools can provide accessible, culturally responsive mental health support using existing resources.
- Peer mentorship offers a scalable and affordable strategy to advance mental health equity for refugee youth.

**Abstract:**

Children from refugee backgrounds in the United States experience significant mental health disparities resulting from cumulative exposure to war-related trauma, forced displacement, and post-migration stressors. Elevated rates of post-traumatic stress disorder, depression, anxiety, behavioral difficulties, and social isolation are well documented, yet access to timely and appropriate mental health care remains limited. In the recent political climate, the immigration enforcement policies create additional fear, instability, and trauma for refugee families. This disproportionately threatens psychological wellbeing, as well as harmony among school and healthcare systems. Structural barriers including language differences, stigma, financial constraints, limited cultural competence among providers, and shortages in the mental health workforce continue to impede care and exacerbate long-term psycho-social inequities. Emerging evidence supports trauma-informed, culturally responsive, and community-based interventions as effective strategies to reduce symptoms and promote resilience among refugee youth, particularly when delivered in accessible settings such as schools and community centers. Family-based and peer-led approaches have shown promise in strengthening social support, fostering belonging, and supporting long-term adjustment. This perspective synthesizes current evidence on mental health intervention models for children from refugee backgrounds and highlights persistent gaps related to sustainability, scalability, and cost effectiveness. The proposed model emphasizes cultural responsiveness, trauma-informed practices, and low-cost implementation to enhance feasibility and sustainability. Early, school-centered interventions that integrate peer support, family engagement, and multi-system collaboration represent a promising and pragmatic approach to advancing mental health equity for children from refugee backgrounds in the United States.

## 1. Background

Children of refugees are defined as minors whose parents experienced forced displacement and resettlement. Refugee children in the United States experience significant mental health disparities resulting from cumulative exposure to war-related trauma, forced displacement, and post-migration stressors. Both refugees and children of refugees face unique psychosocial and environmental factors that may impact their well-being. Therefore, this paper will refer to “children from refugee backgrounds” or “refugee youth” as more inclusive terms. Children from refugee backgrounds in the United States face disproportionate mental health challenges due to the compounding effects of war, displacement, and resettlement stressors. These children exhibit higher rates of post-traumatic stress disorder (PTSD), depression, anxiety, behavioral disorders, and social isolation when compared to their U.S.-born peers [1,2]. Mental health outcomes are further influenced by pre-migration trauma, family separation, disrupted education, poverty, and cultural adjustment difficulties [2,3]. Additionally, heightened immigration enforcement in the current political environment has increased uncertainty and concerns about safety among refugee families [4]. Beyond specific policies, a broad antiimmigrant rhetoric and exclusionary social climate can further exacerbate children’s emotional well-being by contributing to chronic stress, fear social isolation and diminished feelings of belonging. This complicates the development of trust with schools and healthcare providers.

**Figure 1:**
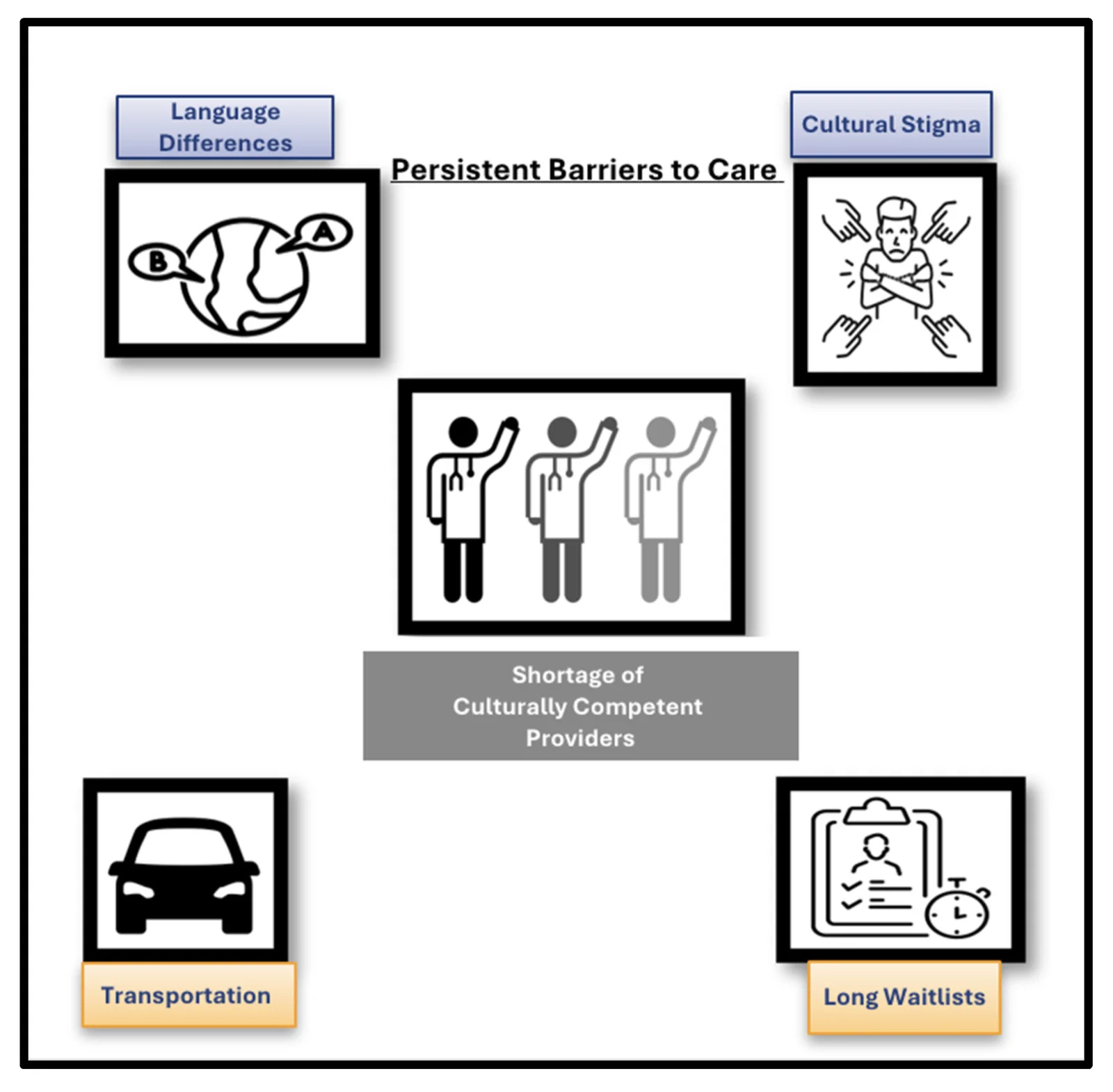
Persistent structural barrier models obstructing access to mental health care services.

Despite the severity of these issues, access to appropriate mental health care remains limited. Structural barriers such as language differences, stigma, financial constraints, and a lack of culturally competent clinicians continue to obstruct care [5]. Figure 1 displays the many factors preventing proper care for children from refugee backgrounds in the United States. These limitations not only delay treatment but also perpetuate long-term disparities in mental health and psychosocial outcomes among refugee youth in the United States [6,7].

Emerging evidence supports the effectiveness of trauma-informed, community-based, and culturally responsive interventions in reducing symptoms and promoting resilience [8,9]. Interventions delivered in accessible settings such as schools, homes, and community centers have shown improved emotional regulation, peer relationships, and overall well-being [10,11]. Furthermore, family-based and peer-led approaches are increasingly recognized for their ability to strengthen resilience and facilitate long-term adjustment [12]. Table 1 demonstrates the various interventions currently available for refugee populations as well as the subsequent cost and benefits of each.

However, gaps remain in the literature regarding the cost effectiveness, sustainability, and the long-term impact of these models. A more integrated, multisystemic approach is urgently needed. Approaches addressing both the psychological and structural determinants of mental health and advocates for equitable access to early, evidence-based support services [13]. Recent research further underscores the importance of addressing refugee children’s mental health through multisystemic and culturally grounded approaches. Systemic barriers, including language differences, provider shortages, and immigration-related stigma, continue to compound the mental health burden for refugee youth in the U.S. and inhibit equitable access to care [13,14]. These challenges are especially pressing for unaccompanied minors and children facing complex trauma, necessitating more specialized and community-engaged interventions.

**Figure 2:**
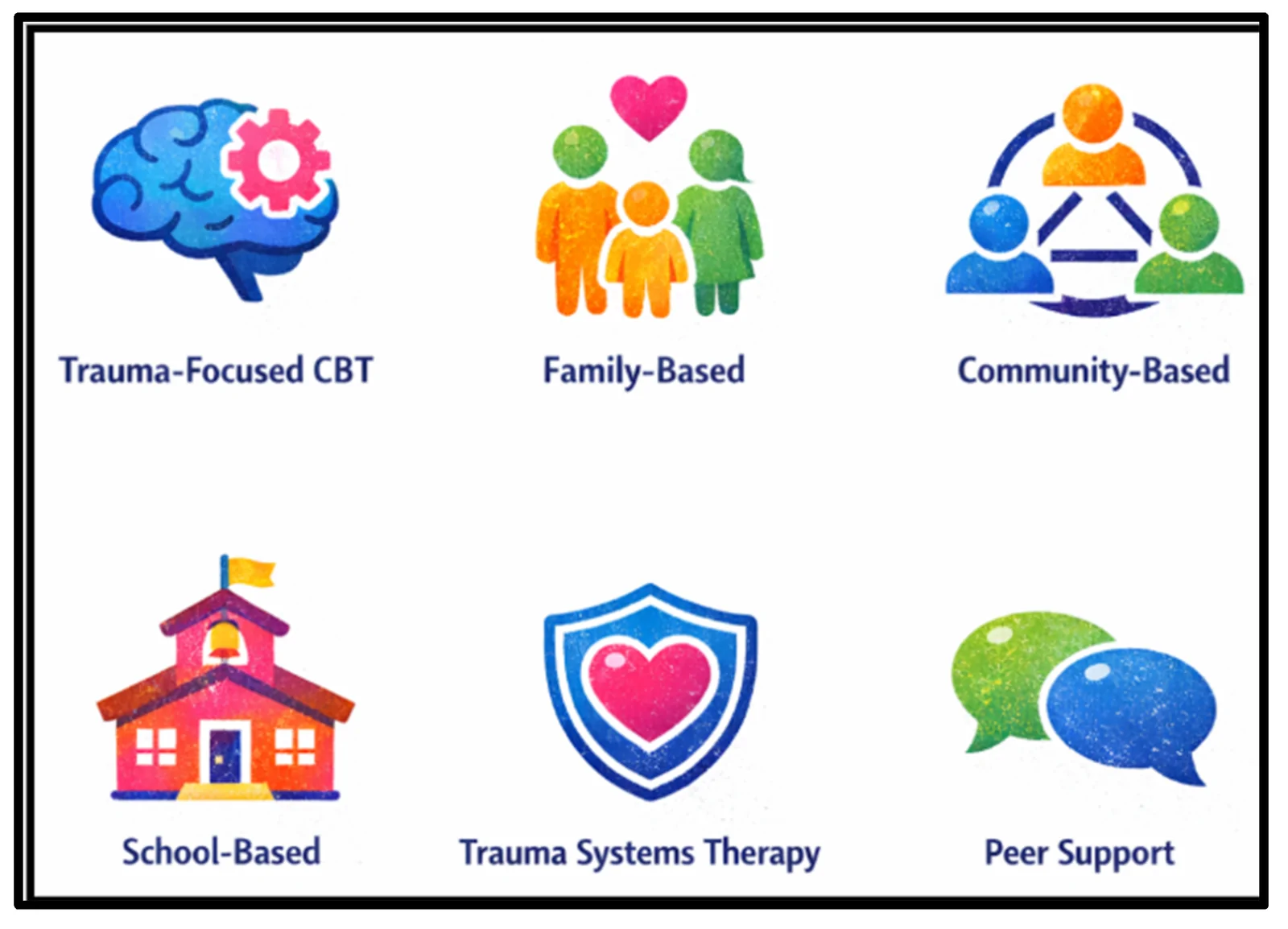
Broad area categories of trauma-informed and culturally competent interventions.

Figure 2 illustrates evidence-based support for a resilience-based framework, emphasizing the protective role of strong family ties, cultural identity, and psychosocial support systems in mitigating the adverse effects of war and displacement [12]. An evidence-based strategy that has proven successful included family-based interventions which significantly improved mental health outcomes when incorporated with caregiver support, culturally relevant practices, and trust-building with healthcare providers [15,16]. Similarly, prior research points towards the benefits of embedding mental health care in accessible, community-based settings, such as schools and community centers, in meeting the linguistic and cultural needs of refugee families as well as providing trauma-informed care [12,15,16].

## 2. A Peer Mentorship and Support Program to Address the Issue

To address the mental health challenges of children from refugee backgrounds in U.S. schools, this intervention proposes the implementation of a Peer Mentorship and Support Program (PMSP). This is a multi-tiered prevention program, which provides universal, proactive interventions designed to promote health or well-being and prevent problems before they occur across an entire population. In this case, this includes a trauma-informed, culturally responsive initiative designed to foster belonging, resilience, and emotional well-being among children from refugee backgrounds in U.S. schools. This strategy is grounded in research indicating that peer relationships can mitigate feelings of isolation, enhance school engagement and performance, improve social and language skills, and encourage community engagement [17]. Additionally, we propose that this can also be considered a prevention program that can be targeted to students who need additional support in social and academic settings. The proposed program pairs refugee students with trained peer mentors and integrates structured support group sessions to encourage social connection, identity development, and the normalization of help-seeking behaviors. Ideally, the peer mentors would be older students with similar experiences.

Research has shown the importance of community resources for protecting the mental health of children, especially those at risk for poor mental health [8]. Similarly, a child’s educational setting can provide protective factors for mental health, including peer mentoring. Many improved outcomes can be linked to the implementation of a peer mentorship program for children from refugee backgrounds, such as improved confidence, resilience, and hope [18,19]. Several studies have shown the benefit of including peer mentorship programs in an educational setting for children from refugee backgrounds, noting increased resilience and empowerment of the students through the feedback of peer mentors [18,20]. Similarly, growing research shows that the implementation of culturally competent and trauma-informed peer programs can have greater benefits compared to current models of mental health support in schools [18].

The main program’s objective would be to reduce emotional distress and social isolation among children from refugee backgrounds. In turn, these students will enhance their coping skills, resilience, and help-seeking behaviors as they adjust to their environment. The program would involve students volunteering to be peer mentors. Student volunteers would be matched with a student looking to join the program or recommended by their teacher in need of a peer mentor. Ideally, students would be matched to a peer mentor of a similar experience background. This would allow stronger connections to be built through trust and vulnerability. The PMSP would involve weekly after school meetings between the peer mentor and student as well as regular check-ins. All components of the program are designed to be linguistically accessible and culturally responsive [18,20]. Participation would be voluntary, and student privacy would be protected through informed consent and confidentiality protocols.

Similarly, a school-based approach that could effectively support the mental health needs of children from refugee backgrounds is a support program integrated into existing school structures [21]. This low-cost, trauma-informed program is designed to utilize current resources and community partnerships to provide culturally responsive mental health support, peer connection, and family engagement. This program would utilize existing school staff, such as social workers, guidance counselors, and English as a Second Language (ESL) teachers. These teams would receive basic trauma-awareness training during professional development days to enhance their ability to identify and respond to students’ emotional needs [18]. Instead of hiring new staff, the program would emphasize coordination among current personnel to offer targeted services, such as informal counseling, small group check-ins, and social-emotional learning activities, integrated into classroom routines.

A core element would be sustained through teacher and parent volunteers and led by older student “cultural ambassadors” (bilingual students or those with shared cultural backgrounds), all of whom will receive peer support training and work under the guidance of a school wellness team. These readily available, peer-led groups would provide a safe space for refugee students to build social connections, reduce isolation, and foster belonging. Teachers could integrate these sessions into advisory periods or after-school programming.

Family Wellness nights held monthly would rely on volunteer support from bilingual parents, community members, and school staff. These events would include informal discussions, basic wellness education, and translation services, helping to build trust and improve communication between schools and refugee families Additionally, art therapy can be incorporated into the PMSP as a creative activity that gives children a comfortable and safe, nonthreatening approach to express their emotions and experiences [22]. Activities such as drawing, painting, or creating magazine collages can encourage self-expression and stress reduction while helping children build coping skills and providing an opportunity for peer interactions. Overall, the innovation of this PMSP resides in the combination existing peer mentorship program protocols within existing school infrastructures for children from refugee backgrounds in the United States. Additionally, this initiative utilizes cultural ambassadors and family involvement to expand previously demonstrated successful techniques while incorporating new proven methods of success [18,22].

## 3. How Can a PMSP Be Implemented

PMSP can be implemented in four phases, beginning with a needs assessment to tailor the intervention to the specific cultural and psychosocial needs of refugee students. A planning committee comprising school personnel, school counselors, and student peer mentors will guide program development. The initiative will recruit upper-grade students as peer mentors through an application process, prioritizing those with refugee or immigrant backgrounds. Selected mentors will complete a trauma-informed training curriculum focused on cultural sensitivity, communication, and confidentiality, which will ensure proper planning and implementation [23]. These students can receive volunteer hours or credits towards their graduation requirements. Concurrently, refugee students identified by school counselors or ESL staff will be invited to participate as mentees, with consent obtained from their guardians.

Mentors and mentees will be paired for regular one-on-one meetings, and small-group support sessions will be held weekly during the school day. These sessions, facilitated by a trained school counselor, will center on emotional regulation, acculturation of stress, identity development, and resilience-building through discussion, and creative expression. School personnel would play an active supervisory role throughout implementation, including mentor selection, training, oversight of peer interactions, identification of students requiring additional support, and referral to appropriate services when concerns arise.

Evaluation will be conducted using pre- and post-intervention Strengths and Difficulties Questionnaire (SDQ) questionnaires measuring the teacher’s assessment of each student involved in the program after one semester. In addition to the SDQ, teachers will complete a separately developed 10-item exploratory assessment designed to capture observable indicators of social integration, peer engagement, and school adjustment.

This questionnaire is presented in Table 2. Each question will be rated on a 0–5 scale, assessing areas such as engagement, language and communication skills, social connection, confidence, emotional well-being, resilience, coping, and help-seeking behaviors. Each question is reflected as: 0 = None; 1 = Very Low; 2 = Low; 3 = Moderate; 4 = Good; 5 = Strong. Scores from all 20 questions will be averaged to create an overall pre-program and post-program score. The percentage improvement will be calculated using the following formula: [(sum of post-program average for all students − sum of pre-program average for all students) ÷ sum of pre-program average for all students] × 100. A 20% or greater increase in the overall average score will be considered the program’s benchmark for effectiveness, indicating meaningful improvement in the outcomes targeted by the PMSP.

Ongoing adjustments will be informed by student input and mentor debriefings. To ensure sustainability, mentor training may be integrated into broader school leadership programs, with materials developed for replication across schools serving refugee populations. Similarly, to promote the continued success of this program, prior mentees will be encouraged to apply for mentor roles to aid new refugee students in their acclimation process, as this can promote leadership skills and broaden social networks [24].

The school-based wellness and support strategy can be phased in using existing infrastructure to minimize financial strain and maximize impact. The first step involves identifying and organizing a school wellness team composed of current staff, such as social workers, guidance counselors, ESL teachers, and interested educators, who already interact with refugee students. These individuals can participate in trauma-awareness and cultural competency training during scheduled professional development days.

As guidance, a peer mentoring manual will be utilized to appropriately fit the needs of students. This manual provides a framework for the training modules involving emotional regulation and self-soothing activities, group discussions, connecting across cultural values, and providing additional resources to families [23]. Once trained, the wellness team can begin offering informal counseling, check-ins, and classroom-based social-emotional learning activities. These can be embedded into the school day through homerooms, advisory periods, or existing support blocks. Coordination among staff ensures that students receive consistent, compassionate care without requiring new hires or major schedule changes.

To promote peer support, older students who share cultural or linguistic backgrounds can be identified and invited to serve as “cultural ambassadors.” These students would receive basic peer mentorship training from school staff or partner organizations. Teachers could host weekly peer support circles during advisory periods or after school, creating inclusive spaces where refugee students can connect, share experiences, and build relationships.

Involving families is equally essential to this program to further support the students [12,19]. Schools can organize monthly Family Wellness Nights using existing spaces, including cafeterias, classrooms, or libraries. With help from bilingual parents and teacher volunteers, these events can offer translation, wellness information, and open discussions that empower caregivers.

Finally, schools should compile a referral directory of local mental health and social services that are affordable and culturally competent and can be listed in the manual [23]. Wellness teams can then guide families toward appropriate external support when more intensive or long-term care is needed. By grounding the program in existing resources and community collaboration, schools can implement a sustainable, impactful strategy to support refugee students’ mental health and well-being [16].

## 4. How Would a PMSP Be an Expected Cost-Effective Intervention

**Figure 3:**
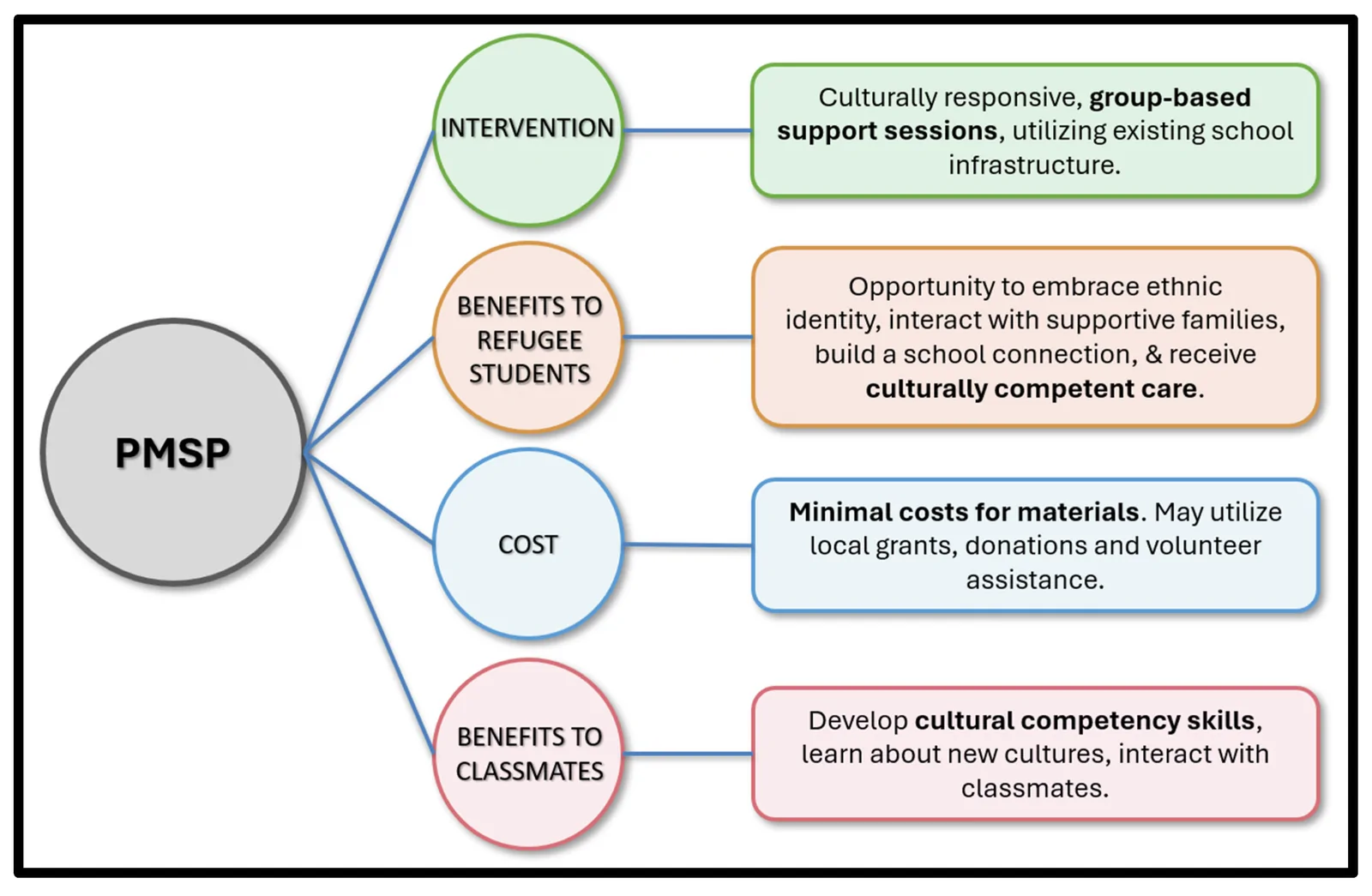
Peer Mentor and Support Program Breakdown. Key findings related to children from refugee backgrounds mental health illustrate how trauma-informed interventions and protective factors support emotional regulation, while also highlighting the barriers to care.

PMSP has the potential to provide a cost-effective approach to addressing the mental health needs of refugee children, particularly when compared to the long-term social and economic costs of inaction [16,20,21]. The program leverages existing school infrastructure and student leadership, while minimizing the need for intensive clinical staffing or external services. By training peer mentors and utilizing group-based support sessions facilitated and supervised by school counselors, the intervention reduces reliance on one-on-one therapeutic services, which are often expensive and underutilized by refugee families due to systemic barriers [25]. In contrast, failing to provide structured mental health support contributes to increased absenteeism, behavioral issues, academic failure, and eventual dropout. These are factors linked to higher public expenditures in healthcare, social services, and juvenile justice in the long term [26]. Evidence suggests that early, school-based mental health interventions not only will improve psychosocial outcomes but also yield a return on investment by preventing more costly interventions later in adolescence and adulthood [27]. Moreover, peer mentors serve to complement rather than replace professional mental health support. As such, the PMSP offers a model that may offer economic advantages relative to more resource-intensive interventions. Peer Mentor and Support Program Breakdown is presented in Figure 3.

The school-based wellness strategy is designed to be affordable and sustainable by using existing resources [21,27]. Current school personnel, such as counselors, social workers, and ESL teachers, can integrate support as part of their regular duties. Trauma-informed training and childcare training can be integrated into existing professional development using free or low-cost resources. Peer-led groups and cultural ambassador programs rely on student volunteers, with minimal materials and staff oversight. Family Wellness Nights can be organized by student volunteers and faculty advisors with additional support from parent and teacher volunteers, reducing the need for outside funding. Activities take place in existing school spaces, which will require no new infrastructure as well as maintaining school supervision via school counselors and/or ESL teachers. If children need serious management of care, school counselors will refer to social workers that can aid in referrals. Any minor costs, such as printed materials or snacks, can be supported through small community grants, or donations. Overall, the program offers an anticipated cost-effective approach that fits within current school structures while addressing refugee students’ mental health needs [17,26]. While school-based interventions cannot eliminate broader structural and sociopolitical stressors affecting refugee families, they may provide an important source of stability, support, safety, and community connection for children navigating these challenges.

## 5. Optional Activities for Family Engagement

In terms of art therapy as an activity to include in the PMSP, studies have demonstrated that participation in expressive arts therapy is associated with significantly increased life satisfaction alongside reductions in mental health symptoms among children from refugee backgrounds [23,24,28]. These programs not only provide therapeutic relief but also equip children with vital coping strategies to manage stress and mitigate symptoms related to past trauma [28]. This underscores the broader significance of a supportive post-migration environment for refugees, particularly those with pre-migration experiences of severe trauma and human rights violations.

The integration of art therapy offers a comprehensive layout for addressing the mental health needs of children from refugee backgrounds. Programs, such as art therapy, acknowledge the diversity of individual preferences and promote cultural humility for specific forms of artistic expression. By doing this, creative art therapy remains both fluid and trauma responsive, thereby enhancing its effectiveness across diverse refugee experiences [24,28]. One meaningful way to do this is by creating spaces where individuals feel safe enough to express themselves, especially when traditional communication is hindered by fear, trauma, or language barriers.

## 6. Conclusions

School-based interventions can provide a novel, potentially cost-effective, and sustainable model for children from refugee backgrounds entering the United States. The Peer Mentorship and Support Model provides community support through a culturally competent lens while addressing the dynamic needs of an underserved population. Existing school structures can be utilized to provide trauma-informed care for children from refugee backgrounds. Addressing the mental health of refugee children early in the transition period allows for earlier prevention of psychosocial issues, leading to greater strain on the healthcare system. In summary, we expect this could be a promising and potentially cost-effective approach that could provide significant benefits to refugee populations across the United States. Future research is needed to evaluate the feasibility, effectiveness, and cost-effectiveness of the PMSP in real-world school settings.

## Figures and Tables

**Table 1:** An overview of mental health intervention approaches for children from refugee backgrounds, highlighting their core characteristics, benefits, and challenges to implementation.

Intervention Type	Description	Key Benefits	Challenges
Trauma-Focused Cognitive Behavioral Therapy	Structured, evidence-based therapy that helps children process trauma & reduce symptoms	-Reduces PTSD, depression, & anxiety -Strengthens coping skills & resilience	-Requires trained therapists -Not culturally adaptable for all refugee groups
Family-Based Interventions	Engaging interventions for refugee children and their families strengthen family bonds & improve mental health	-Enhances family support systems -Promotes long-term resilience through family cohesion	-Disrupted family dynamics -Requires family involvement, which can be challenging
Community-Based Interventions	Community-driven programs that provide mental health support via local services, schools, & peer groups	-Lowers stigma -Increases accessibility -Builds community support and social networks	-Lack of trained professionals in community settings -Limited funding for sustainability
School-Based Mental Health Programs	Mental health support is provided in schools, focusing on peer support, social-emotional learning, & trauma-informed practices	-Integrates mental health support into children’s daily environment -Reduces stigma -Increases early intervention	-Lack of mental health training -Requires resources & long-term commitment
Trauma Systems Therapy for Refugees	A multi-tiered, culturally responsive intervention that targets trauma & migration-related stressors, using community-based settings	-Tailored to refugee populations -Addresses systemic barriers -Builds community trust	-Resource-intensive -Requires ongoing community collaboration & funding
Peer Support Programs	Peer programs that focus on shared experiences as an aim to reduce isolation & promote resilience	-Builds peer networks -Reduces social isolation -Encourages shared experiences & emotional support	-Inconsistent peer dynamics -May require training for peer leaders

**Table 2:** Intervention Effectiveness Pre and Post Teacher-Completed Exploratory Assessment (10 items). Scoring scale: 0 = Not at all; 1 = Very low; 2 = Low; 3 = Moderate; 4 = Good; 5 = Great.

1. How engaged is the student in classroom activities?	0 1 2 3 4 5
2. How comfortable is the student interacting with peers for leisurely activities?	0 1 2 3 4 5
3. How comfortable is the student communicating with peers?	0 1 2 3 4 5
4. How well does the student communicate with teachers and school staff?	0 1 2 3 4 5
5. How would you rate the student’s English language skills?	0 1 2 3 4 5
6. How confident is the child when expressing their thoughts, feelings, or needs?	0 1 2 3 4 5
7. How effectively does the child cope with stress or difficult situations?	0 1 2 3 4 5
8. How willing is the child to seek help when they need it?	0 1 2 3 4 5
9. How would you rate the child’s overall emotional well-being?	0 1 2 3 4 5
10. How comfortable is the child expressing their cultural identity at school?	0 1 2 3 4 5

Note: The SDQ is a standardized measure administered separately from the exploratory 10-item teacher-completed assessment proposed for this program.

## Data Availability

No new data was created for this manuscript. Data sharing is not applicable to this article.
